# Danish doctors’ reactions to ‘internationalization’ in clinical training in a public university hospital

**DOI:** 10.1186/s13104-019-4405-y

**Published:** 2019-07-15

**Authors:** Joyce Kling, Martin G. Tolsgaard, Ellen Løkkegaard, Grete Teilmann, Gylli Mola, Jørgen Hedemark Poulsen, Lisbeth Nilas, Dina Cortes

**Affiliations:** 1 Centre for Internationalisation and Parallel Language Use, University of Copenhagen, Emil Holms Gade 4, 2300 Copenhagen S, Denmark; 2 Faculty of Health and Medical Sciences, University of Copenhagen, Copenhagen, Denmark; 3 Department of Obstetrics and Gynecology, University of Copenhagen Hospital North Zealand, Hillerød, Denmark; 4 Copenhagen Academy for Medical Education and Simulation (CAMES), Rigshospitalet, Copenhagen, Denmark; 5 Department of Pediatrics, University of Copenhagen Hospital North Zealand, Hillerød, Denmark; 6 Section of Quality of Education and Management Information, Studies and Student, Faculty of Health and Medical Sciences, University of Copenhagen, Copenhagen, Denmark; 7 Department of Obstetrics and Gynecology, Copenhagen University Hospital Hvidovre, Hvidøvre, Denmark; 8 Department of Pediatrics, University of Copenhagen Hospital Hvidovre, Hvidovre, Denmark

**Keywords:** Clinical teaching, Internationalization, Teaching in foreign language, English medium instruction

## Abstract

**Objective:**

From 2012 to 2015, two Departments of Obstetrics and Gynecology and two Departments of Pediatrics at the University of Copenhagen implemented an English medium international project. The project allowed international students to work in pairs with local Danish speaking students in a clinical setting. The student cohort was supported by Danish doctors who were responsible for student-pair supervision in English and, ultimately, patient care. Drawing on survey responses of 113 Danish doctors, this study considers the doctors’ overall evaluation of the program and their perception of the international students’ knowledge, skills and attitudes compared with local students.

**Results:**

The Danish doctors rated the international and local students comparable in respect to professional commitment (p = 0.347), academic level (p = 0.134), and English proficiency (p = 0.080). The Danish doctors rated the international students significantly lower than the local students regarding communication with Danish doctors, other hospital staff, and patients (p < 0.001 in all cases). Ninety percent of the doctors involved in the project supported continuing working with internationalization if it included mixed pairs of students and a Danish doctor assigned each day to be exclusively responsible for student supervision. Language barriers for international medical students could be overcome but required substantial faculty support.

**Electronic supplementary material:**

The online version of this article (10.1186/s13104-019-4405-y) contains supplementary material, which is available to authorized users.

## Introduction

Increased globalization of higher education with rapid dissemination of knowledge has spread into the medical education arena [1]. One specific aspect of this has been an increase of English medium instruction across Europe at the tertiary level [2]. As the majority of students and lecturers in these English medium instruction courses are non-native speakers of English, these linguistic challenges are often underestimated [3]. The literature contains some reports of inclusion of non-native English speaking students in medical courses in Anglophone countries [4] and a move toward internationalized English in medical education and global health education [5]. However, little has been published on internationalization of clinical training of international students in non-Anglophone countries where English is neither the standard language of choice for medical education nor patient treatment. Historically, enrollment of international exchange students in medical education courses has been limited to isolated courses that have been running in English designed for a guest population with limited access for and interaction with local students and patients. This solution to internationalization often led to dissatisfaction on the part of both teachers and students [6].

This paper focuses on a project developed to adjust for this situation by providing opportunities for inclusion of both local and international students in an innovative training program. In the following, we present doctors’ overall evaluation of this English medium instruction project and their perceptions of international students’ knowledge, skills, and attitudes compared with local students in a Danish context.

## Context

As part of its internationalization strategy, The University of Copenhagen promotes collaboration and partnerships. At the Faculty of Health and Medical Sciences, international study exchange of students primarily takes place through the Erasmus and Nordplus programs, which are based on bilateral agreements [7]. To increase local Danish students’ exposure to an internationalized curriculum and include non-Danish speaking students who speak English as a foreign language in the local context, the University of Copenhagen implemented English medium instruction clinical training courses in Obstetrics and Gynecology and Pediatrics. While the implementation of this educational protocol resulted in improved student evaluations of the international medical program at University of Copenhagen [7], it also resulted in a range of unforeseen challenges for the doctors in regard to English language use. Doctors in this program rated teaching in English as 30% more difficult than in Danish, and a subgroup of doctors had difficulties in all forms of communication in English in this non-Anglophone hospital setting [6].

The international medical program was linked to two 5-week clinical course in Obstetrics and Gynecology, and Pediatrics, respectively, which were offered in the final semester of the University of Copenhagen master’s degree program. Four departments at the two University of Copenhagen teaching hospitals (Hvidovre and North Zealand Hospital) participated in the project. The students worked in pairs (one local Danish speaker and one international non-Danish speaker) on the wards and in the clinics, supported by assigned attending doctors who exclusively taught the students and were responsible for the patients of the pairs of students. Each day, one doctor was appointed wholly responsible for teaching and overseeing the student pairs in the clinic. Lectures and meetings primarily took place in English, with translation and interpretation support in the clinic and on the ward from local students for activities that took place in Danish. In exchange for their work with the international students, the local Danish-speaking students received supplemental certification.

## Main text

### Methods

An original questionnaire was locally developed in Danish by the Section of Quality of Education and Management Information, Studies and Students at the University’s Faculty of Health and Medical Sciences to study to measure the doctors’ attitudes regarding participation in the program described above (see Additional file 1: Questionnaire). A paper-based version of the questionnaire was distributed at a morning meeting. The doctors responded to 27 questions related to the program’s format overall, as well as the students’ academic performance, academic engagement, overall general English proficiency, and professional communicative competence with doctors, hospital staff and patients. The questions were evaluated on a 7-point Likert scale with rating of 1 = unacceptable, 4 = acceptable and 7 = excellent. The questionnaires also included open-ended response questions.

Quantitative data were analyzed using the non-parametric tests, as the data were not normally distributed based on histograms. We used Mann–Whitney test, Fisher’s exact test and Chi-square test with double-sided p-values and a significance level of < 0.05.

The open-ended qualitative responses were transcribed verbatim, translated into English, and coded using thematic analysis by one member of the research team. These data were then independently reviewed by a second member of the research team. The coded themes and sub-themes were compared and agreed upon by the two researchers. These themes were subsequently analyzed, and conclusions were determined.

According to Danish law and the regulations of the regional ethics committee, survey studies are not required to apply for ethical approval. Thus, this study was not sent for ethical review.

### Results

Of a total of 205 possible doctors at the four departments, 113 surveys were collected; a response rate of 55% (113/205). Responses were anonymous and did not influence teaching assignments or responsibilities.

As Table 1 shows, the doctors rated the international students and local students to be comparable in respect to professional commitment, academic level, and English proficiency. In contrast, the doctors rated the international students significantly lower than the local students in communication with the doctors, other hospital staff, and patients, (p < 0.001 in all cases). However, the doctors found communication of the international students to be acceptable 94% of the time, 88% of the time, and 78% of the time, with the doctors, the staff, and with the patients, respectively.

**Table 1:** Doctor’s ratings of aspects of the international teams in clinical training program

	About international students	About Danish students	p-values
Response-rate	55% (113/205)	55% (113/205)	
How do you rate the students’ professional commitment?	5 (2–7), n = 85	6 (3–7), n = 97	0.347
How do you rate the students’ academic level?	5 (3–7), n = 80	5 (3–7), n = 97	0.134
How do you rate the students’ level of English?	6 (3–7), n = 94	6 (4–7), n = 87	0.080
How do you rate the communication between the students and doctors?	5 (3–7), n = 93	6 (4–7), n = 94	< 0.001
How do you rate the communication between the students and other staff?	3.5 (2–7), n = 66	6 (3–7), n = 80	< 0.001
How do you rate the communication between the students and patients?	4 (1–7), n = 72	6 (4–7), n = 83	< 0.001

Median (range) rates. The questions were evaluated on a 7-point Likert scale with rating of 1 = unacceptable, 4 = acceptable and 7 = excellent

Table 2 conveys the doctors’ personal reactions to working in the program. Fourty five percent of the doctors had worked with students who trained in pairs (a Danish and a non-Danish speaking student). More doctors at the departments of Pediatrics than at the departments of Obstetrics and Gynecology had worked with students who trained in pairs, (p < 0.0001). Overall, 90% of the doctors who had worked with pairs of students reported giving feedback to the students involved. These respondents rated this experience as good, at a median of 6 (range 3–7) with, only 2% (1/50) rating this unacceptable.

**Table 2:** Doctors’ experience working with International teams consisting of a Danish speaking and non-Danish speaking students

	International teams, in total	International teams at Departments of Obstetrics and Gynecology^a^	International teams at Department of Pediatrics^b^	p-values
Have you worked with students who trained in pairs?	45% (51/113)	25% (13/53)	63% (38/60)	< 0.001^d^
If you worked with students who trained in pairs, how do you rate this?	6 (3–7), n = 50	6 (3–7), N = 13	6 (4–7), N = 38	0.542^c^
If you worked with students who trained in pairs, did you give feedback to the students?	90% (43/48)	92% (12/13)	90% (34/38)	0.808
If you have given feedback to a pair of students, how do you rate this?	6 (3–7), n = 46	6 (3–7), N = 12	6 (4–7), N = 35	0.529^c^
Doctors who support that the departments continue with the international teams	90% (92/102)	94% (46/49)	87% (46/53)	0.385

Median (range) rates. The questions were evaluated on a 7-point Likert scale with rating of 1 = unacceptable, 4 = acceptable and 7 = excellent

^a^The meetings were in English

^b^The majority of meetings was in Danish

^c^Mann–Whitney test done by http://www.socscistatistics.com/tests/mannwhitney/Default2.aspx

^d^Yates corrected Chi square test done by http://www.openepi.com/Menu/OE_Menu.htm

In total, 90% of the responding doctors supported continuing working with the program. There was no difference between the results from the departments of Pediatrics and of Obstetrics and Gynecology.

The results of the qualitative responses fall into three themes: challenges related to program design, language, and teaching (see Table 3). In general, the doctors supported the program, but overall expressed that they found it essential that the students worked in pairs to support each other, and that there was a doctor assigned each day to work exclusively with them. Some doctors also desired an additional nurse for the pairs of students.

**Table 3:** Questionnaire to the clinical Danish doctors at the Departments of Obstetrics/Gynecology and Pediatrics

General themes	Sub-theme
Program design	Additional staffing required to accommodate English medium instruction out-patient clinic—one doctor must be exclusively allocated each day for international teams Scheduling must accommodate pairs of students in the clinic and on the wards Academic requirements differ at partner universities
English as the language of instruction	Opportunity to develop medical and academic English proficiency Pair work (translation) mandatory for interaction with patients Limitation for learning for the international students since patient history is often told in Danish Reduced number of critical questions at meetings Reduction of number of spontaneous discussions Occasional problems for students at examinations due to cultural/linguistic differences
Teaching	Additional time for preparation and feedback required for English medium instruction Inspiration and insights from the international students

As for the challenges related to program design, doctors noted that if left on their own, international students could be a burden. Although the local students were able to translate for their international classmates when meeting with patients and on the ward, responsibility for accurate dissemination of content, patient history, and ultimate care remained with the doctors. The doctors needed to replicate the input to communicate and assist those students who could not follow along on their own because of either language barriers or differences in formal academic training. Comments noted additional stress and responsibilities in relation to scheduling when working with international student on the ward and in the clinics., e.g., “it was difficult when the Danish student was not present and the doctor had the international student alone” (respond no. 33) and “it was difficult to have the pair of students on the ward, because there sometimes was lack of time” (respondent no. 4). Some doctors also noted that difficulties in recruiting patients for clinical training because they felt uncomfortable with a non-Danish speaking person participating in the consultation.

Regarding the use of English as the language of instruction, comments included both negative and positive responses. For example, doctors reported that they found the need of translation between students and between students and patients stressful. The same was stated about prevailing general linguistic challenges of working in English as a foreign language. Doctors’ noted that the linguistic shortcomings due to limitations in the English competences led to more superficial academic discussions during the meetings and often a lack of spontaneous comments and opinions, e.g., “the teaching is not as detailed as it is in Danish” (respondent no. 10) and reduced inclination to initiate discussions on patient treatment e.g., “it is not as detailed, and you get the impression that not everyone contributes when it is in English” (respondent no. 14).

In terms of teaching, the responses noted that international students provided an opportunity to be part of an international environment, to learn from others and to practice speaking English. The doctors mentioned that once past an initial learning curve, the effort of working with the international students had been rewarding, e.g., “It is undoubtedly a lot more stressful/challenging for the individual doctor and the department’s other staff to have international students than Danish students. However, the international students are also very sweet and interested, as well as a breath of fresh air” (respondent no. 74).

### Discussion

This study showed that Danish doctors found it was a challenge to let international non-Danish students work with Danish speaking patients. However, 90% of the doctors supported continuing the program contingent on the international students working in pairs with Danish speaking students in the clinical setting, and doctors specifically assigned to work in English with the students and be responsible for both student-pair supervision and patient care.

Although the doctors rated the students’ English proficiency and communication between doctors and students acceptable (Table 1), the qualitative data showed the linguistic challenges caused some concern, primarily in terms of nuanced discussion of patient care in English. Ultimately, for clarity and expedience, clinical conferences reverted to the doctors’ first language, Danish. Many of the doctors’ comments focus on these challenges and highlight concerns from a pedagogical perspective that mirror previous English medium instruction research [8]. However, allowance for simultaneous parallel code use [9] and the flexibility of English as a lingua franca [10] provided some assistance in the multilingual, multicultural learning setting. While the linguistic challenges of English medium instruction were expressed in these early stages of implementation, as documented in previous research [11–13], these challenges began to ease with additional English medium instruction teaching experience.

While previous literature claims that pair training may not be applicable to clinical training involving real patients [14, 15], those doctors involved in the authentic clinical training with paired teams in this project found it possible both to take care of the patients and to teach the pairs of students. Moreover, the students appreciated working in these pairs [7]. An additional element of this program for international students was also the inclusion of treatment of Danish patients and direct contact between the international students and these patients. As this interaction required the assistance of the local Danish speaking students, another valuable outcome of the project was the extensive interaction Danish students experienced from working in English in pairs with the international students and the appointed doctors who worked with the teams of students.

### Conclusion

In this Danish study, we found language communication barriers for non-native speaking international medical students could be overcome, but substantial faculty support was needed to ensure well-functioning clinical clerkships. Nonetheless, the majority of the supervising doctors were highly supportive of teaching in English given that additional faculty support was provided.

## Limitations

The response rate for the survey was 55%, which may mean that we cannot rule out responder bias. However, it is likely that a substantial fraction of the 45% non-respondents had not been personally involved in the teaching of international students. Moreover, only 45% of the responders had worked with these pair of students. Finally, it was not possible to do in-depth qualitative interviews, which could have provided richer data on why and how international students were perceived as more resource-intensive to supervise than local Danish speaking students. Lacking this data, we had to rely on those doctors who responded to the open-ended questions.

## Additional file

**Additional file 1.** This is an original questionnaire developed for this study. The questions have been translated from Danish into English.

## Data Availability

The datasets used and/or analysed during the current study available from the corresponding author on reasonable request.
