# Supplementary material for: Danish doctors’ reactions to ‘internationalization’ in clinical training in a public university hospital
Source: BMC Res Notes. 2019 Jul 15;12:411. doi: 10.1186/s13104-019-4405-y (PMC6632207; doi:10.1186/s13104-019-4405-y)
Supplement: Supplementary file 1 — Additional file 1. This is an original questionnaire developed for this study. The questions have been translated from Danish into English. [file 13104_2019_4405_MOESM1_ESM.pdf]

Additional file 1_Questionnaire

Questionnaire on students who trained in pairs with a mix of international and Danish students, Spring 2015.

Dear Colleague,

We have now had training with teams of international and Danish students for a period. We would like to request that you respond to a short questionnaire regarding your experiences with and opinions about teaching these students in gynecology/obstetrics and pediatrics.

The questionnaire comprises the following types of questions:

1. Biodata
2. Evaluation of the international students and of the Danish students
3. Language and communication
4. Opinion(s) regarding future courses in English

Thank you in advance for your assistance.

Regards,

The project team for Internationalization at the Departments of Obstetrics/Gynecology and Departments of Pediatrics at Hvidovre Hospital and North Zealand Hospital, Hillerød, as well as the Evaluation Unit and the International Office, Faculty of Health and Medical Science, University of Copenhagen, March 2015.

1. Did you meet exchange students in your department in connection with teaching in Pediatrics or in Obstetrics and Gynecology in Spring 2015?<br>1) Yes<br>2) No
2. What is your educational background?<br>1) Specialist<br>2) Resident in training for specialization in general practice<br>3) Resident in training for specialization in pediatrics or gynecology-obstetrics
3. In which department are you employed?<br>1) Pediatrics<br>2) Gynecology-Obstetrics
4. Did you work with students who trained in pairs, i.e. an international and a Danish student?<br>1) Yes, only in the student clinic<br>2) Yes, both in the student clinic and other departments, e.g., on rounds, reception<br>3) No<br><br>**If you answered no to question 4, skip questions 5, 6 and 7.**

5. If you worked with students who trained in pairs, how do you rate this? (mark with an X)

1. Unacceptable	2.	3.	4. Acceptable	5.	6.	7. Optimal	8. Don't know

6. If you worked with students who trained in pairs, i.e. an international and a Danish student, did you give feedback to the students together?
   1) Yes
   2) No

7. If you gave feedback to students who trained in pairs together, how do you rate this? (mark with an X)

1. Unacceptable	2.	3.	4. Acceptable	5.	6.	7. Optimal	8. Don't know

8. Please provide any comments or suggestions about working with student pairs:

9. How do you assess the INTERNATIONAL students' academic level? (mark with an X)

1. Unacceptable	2.	3.	4. Acceptable	5.	6.	7. Optimal	8. Don't know

10 How do you assess the INTERNATIONAL students' professional engagement? (mark with an X)

1. Unacceptable	2.	3.	4. Acceptable	5.	6.	7. Optimal	8. Don't know

11. Do you have further comments on the participation of INTERNATIONAL students e.g., in the clinic, on the ward, during rounds, during classes, and or during meetings? (mark with an X)

1. Unacceptable	2.	3.	4. Acceptable	5.	6.	7. Optimal	8. Don't know

12. How do you assess the DANISH students' academic level? (mark with an X)

1. Unacceptable	2.	3.	4. Acceptable	5.	6.	7. Optimal	8. Don't know

13. How do you assess the DANISH student's academic engagement? (mark with an X)

1. Unacceptable	2.	3.	4. Acceptable	5.	6.	7. Optimal	8. Don't know

14. Do you have further comments on the participation of the DANISH students in the clinic, on the ward, during rounds, during classes, and/ or during meetings? (mark with an X)

1. Unacceptable	2.	3.	4. Acceptable	5.	6.	7. Optimal	8. Don't know

15. What did it mean for you that the course was taught in English?

16. Did the department's meetings take place in English when exchange students were present?

1) Yes, all
2) Yes, some
3) No

17. What did it mean for you that meetings were conducted in English?

18. How do you assess the NON-Danish speaking INTERNATIONAL students' general ability to communicate in English? (mark with an X)

1. Unacceptable	2.	3.	4. Acceptable	5.	6.	7. Optimal	8. Don't know

19 How do you assess the DANISH speaking students' general ability to communicate in English? (mark with an X)

1. Unacceptable	2.	3.	4. Acceptable	5.	6.	7. Optimal	8. Don't know

20. How do you assess the NON-Danish speaking INTERNATIONAL student's communication with doctors? (mark with an X)

1. Unacceptable	2.	3.	4. Acceptable	5.	6.	7. Optimal	8. Don't know

21. How do you assess the NON-Danish speaking INTERNATIONAL student's communication with the non-medical staff? (mark with an X)

1. Unacceptable	2.	3.	4. Acceptable	5.	6.	7. Optimal	8. Don't know

22. How do you assess the NON-Danish-speaking INTERNATIONAL student's communication with patients /parents and/or relatives? (mark with an X)

1. Unacceptable	2.	3.	4. Acceptable	5.	6.	7. Optimal	8. Don't know

23. How do you assess the DANISH student's communication with doctors? (mark with an X)

1. Unacceptable	2.	3.	4. Acceptable	5.	6.	7. Optimal	8. Don't know

24. How do you assess the DANISH student's communication with the non-medical staff? (mark with an X)

1. Unacceptable	2.	3.	4. Acceptable	5.	6.	7. Optimal	8. Don't know

25. How do you assess the DANISH student's communication with patients / parents and/or relatives? (mark with an X)

1. Unacceptable	2.	3.	4. Acceptable	5.	6.	7. Optimal	8. Don't know

26. As part of the efforts to internationalize and to profile the department and the hospital, do you support implementation English medium instruction courses for international students in the future?<br>1) Yes<br>2) No
26a. What improvements / changes at the Faculty of Health Sciences do you want regarding future course offerings in English for international students?
26b. What improvements / changes from the department / hospital do you want regarding future course offerings in English for international students?
26c. If no, why not?
26d. If no, what conditions would need to be met to persuade you to change your “no” to “yes”?
26e. If you have further comments or suggestions, please state them here:

Thank you for your assistance. ☺
